# Notes and News

**Published:** 1889-06-08

**Authors:** 


### Notes and News.

Collected and Communicated.

The *Nineteenth Century* for June contains a second article from the pen of Mr. Clifford about the "Hawaiians and Father Damien." *Longman's Magazine* contains a common-sense article by Dr. Richardson on "The Health of the Mind." In the *Leisure Hour* Dr. Schofield gives a warning about the increase of "Nervousness and Hysteria," due to the rate of life at which we now live. The *Fortnightly Review* has an article by Dr. Robson Roose on "The Art of Prolonging Life."

The new hospital for infectious diseases erected at Heathcote, near Warwick, for the use of the Mid-Warwickshire sanitary district, at a cost of nearly £10,000, was opened on May 29th by Ald. S. T. Wackrill, J.P., of Leamington, chairman of the Joint Hospital Board. It is just five years since the commencement of the Heathcote Hospital was sanctioned, and the foundation-stone was laid during the Jubilee year. A Washington Lyons disinfecting apparatus has been secured, and the sanitary arrangements carried out on the most approved plans.

The annual report of the Children's Hospital at Dublin, known as St. Joseph's, tells a tale of much suffering relieved. It was the first Irish hospital established for the children, and is the largest institution of the kind in the country; but it wants a chronic ward. The following is the plea put forth: "With the daily increasing demands on its services the want of a chronic ward sadly hampers the utility of St. Joseph's Hospital. To open a chronic ward the one thing needful is money. Space and beds are ready, but until the means of supporting the occupants of the beds be forthcoming the present unsatisfactory state of things must continue. A sum of £150 per annum would suffice for the support of ten such beds, and it must be remembered that the said sum would represent not alone ten chronic cases provided for, but sixty or seventy other cases treated in the general wards. In conclusion, the Sisters of Charity beg to offer to their supporters their very sincere thanks and the gratitude of 4,260 poor children who have received treatment and benefit in the wards of St. Joseph's Hospital since its institution."

There is no end to the Jubilee! News has lately come from Jhind, near Calcutta, of the laying of the foundation-stone of a Jubilee Hospital. The Maharajah performed the ceremony, and made some very pretty remarks about the generous and benevolent Empress of India.

Oswestry Dispensary is financially in a sound position. The balance-sheet shows that there was a balance in hand on January 1, 1888, of £283 12s. 10d. The subscriptions for the year amounted to £69 16s. 6d., the church collections to £35 17s. 8d., the proceeds of the ball to £21 4s., the receipts from baths to £18 13s., which, with the interest allowed by the bank, amounted to £435 16s. 2d. The expenditure amounted to £146 16s. The members are also increasing.

They are about to perform their first execution by electricity in America. We hope the executioners have read some words of Dr. Richardson's, written not long ago in the *Asclepiad*. He says: "With the great induction coil at the Royal Polytechnic, large animals like sheep, and smaller animals like dogs, were stricken into such apparent death by the discharge from a battery of 96ft. of surface, set in cascade, that the most careful examination would often fail to determine whether or not the blow had been fatal. In short, the recoveries that took place after these extreme shocks were so strange and unexpected, I was obliged to give up the practice I had devised, of using electricity as a method for destroying the lives of the lower animals rapidly and painlessly." Even the most revengeful man would not desire that a criminal should be buried alive.

The visit of the Princess Louise to Leicester to open the Children's Hospital passed off well. At Ely Station Her Royal Highness was met by the mayor and Corporation, who presented an address of welcome. The Princess was greeted with much enthusiasm as she drove through Leicester to the hospital. Among those who attended the ceremony was the Duke of Rutland, Lord Howe, the Marquis of Granby, and other noblemen. The streets were gaily decorated, and the day was regarded as a general holiday at Leicester. The wards of the hospital are all furnished and in working order, and presented a pretty spectacle when visited by the gaily-dressed crowd. In the evening, Mr. J. A. Adcock gave a concert in aid of the hospital, which was largely attended, and it was late at night ere Leicester returned to its usual quiet, and, sunk in slumber, forgot the honour of the Royal visit.

The committee of the Sick Children's Hospital in Great Ormond-street have this year tried a new experiment. Instead of holding their annual dinner, which usually brought in about £2,000 to the coffers of the institution, they called its friends to an afternoon meeting at Dudley House, Park-lane. A well-filled room was the result, and doubtless many of those present shared the opinion expressed by the chairman, Lord Aberdare, that it was pleasanter to discuss the wants of the hospital in the beautiful picture-gallery, among the works of art collected by the late Lord Dudley, than after the numerous courses of a long public dinner. In speaking of the value of hospitals, Sir James Paget gave some remarkable statistics about the loss caused to the country by illness. This, he said, amounted annually to eleven million pounds; therefore anything that tended to lessen disease was—putting all questions of philanthropy aside—an addition to the national wealth. Dr. Cheadle spoke in detail of the needs of the hospital, pointing out the necessity for special wards for whooping cough and other diseases that must now be refused admittance. About £13,000 is still required to complete the building, and £2,000 for the maintenance fund.

THE following vacancies are announced this week, the amount of the salary in each case being given after the name of the institution :

**Resident Medical Officers.**

St. Mary's Hospital, Manchester. Medical Officer.—£70, board, &c.
University College, Liverpool. Demonstrator in Physiology. £120.
North-Eastern Hospital for Children. Junior House Surgeon.—£30, board, &c.
City of London Hospital, Victoria Park. House Physician, &c.
Royal United Hospital, Bath. Medical Officer.—£100, board, &c.
Sunderland Infirmary. House Surgeon.—£80, board, &c.
University College, London. Medical Officer.
Great Yarmouth Hospital. House Surgeon.—£90, board, &c.
Gloucester Asylum. Assistant Medical Officer.—£105, board, &c.
Adelaide Hospital, Australia. Medical Officer.—£500, board, &c.
Miller Hospital, Greenwich. Assistant Medical Officer.—£30, board, &c.

**Non-Resident Medical Officers.**

West Riding of Yorkshire. Medical Officer of Health.—£800.
National Hospital, Queen-square. Anæsthetist.
Dental Hospital of London Medical School. Tutor, £40.
National Hospital, Queen-square. Pathologist and Registrar.—£50.

---

PHOTOGRAPHY, though open to all, is still a monopoly with a few, and under such circumstances it is gratifying to notice that a simple and practical textbook has recently been published by Messrs. Perken, Son, and Rayment, entitled "Beginner's Guide to Photography." Such a book as this will do much to popularise an art that cannot be too popular.

---

A SUCCESSFUL little bazaar was held in one of the male wards of the Yeatman Hospital, Sherborne, on the 14th May, in aid of the Child's Cot Fund. In former years there has only been one stall for useful and fancy articles, but this time Miss A. Courtenay Clarke, the sister in charge, instituted two more for flowers and refreshments, which proved a great attraction and source of gain. The town band attended in the afternoon. The sum of £21 was realised, a much larger amount than usual.

---

THE bazaar, held this week at Totnes, in aid of the Cottage Hospital was first started by the children of the town, and though their elders came forward as the idea grew and lent their aid, still to the little people belongs the chief credit of the affair. Are they also responsible for the little prices? Surely never before was there a bazaar where tea could be had for a penny, and where such good value was received for money laid out. Miss Eveline Trist, daughter of the president of the hospital, opened the bazaar.

---

THE Psychical Society lately held a meeting in Westminster Town Hall to put to a practical test the theories of hypnotism so frequently discussed. The son and the daughter of a distinguished barrister and M.P. were the victims of some of the experiments, but the best results were obtained from some boys from a neighbouring school. In one experiment of deferred hallucination, the professor told the patient, who was momentarily in a cataleptic state, that he would wake up in a minute, and that five minutes later he (the professor) and the chairman would be invisible to the boy. Another lad was told simultaneously that he would wake up in two minutes, and that five minutes later he would be unable to see the candlestick and water-bottle on the table. Everything happened as foreshadowed, one boy walking up against and round and round the professor, but unable to see him, and the other searching for the candle, which was just before his eyes.

THE annual meeting of the North-Eastern Hospital for Children was held on Tuesday, when Mr. Joseph Gurney Barclay (chairman), Miss Philips, and Messrs. J. Lister Godlee, W. A. Barclay, Mr. R. C. Ashby, Maurice Rüffer, and E. W. Grimwade were present. The report was adopted, and Mr. Joseph Gurney Barclay was re-elected president. The number of in-patients treated during the year was 709, and that of the out-patients 13,754. Through the kindness of Mrs. Morland, the committee have for many years had the use of a cottage at Croydon as a convalescent home. It was recently found that accommodation must be provided for patients not so far advanced towards convalescence, and the committee therefore closed the home in June last and are now considering what permanent arrangements had best be made. The Samaritan Fund has been carried on with marked success by the Ladies' Committee, who assisted an increased number of cases during the year, though the funds at their disposal were somewhat less than those available in 1887. The committee especially express their appreciation of the labours of the Matron and House Surgeons. The matron has no difficulty in finding probationers, and frequent visits have been paid her by old patients, who, now no longer children, have come to thank her for the benefits they received years ago in the wards under her charge. The committee have been able to clear off the balance of debt on the old building fund, and otherwise reduce their liabilities, owing to the receipt of nearly £1,100 in legacies. Especial regret is expressed at the resignation of Mr. W. L. Barclay, the Treasurer, on the ground of ill-health, that gentleman having filled the position since 1871. Mr. J. Lister Godlee acts as treasurer *pro tem.* We must congratulate the secretary, Mr. Alfred Nixon, on his successful reduction of the administration expenses, which now stand 10 per cent. lower than formerly, and also for issuing so excellent a report, full of valuable information, and showing a progress in the condition of the North-Eastern Hospital which is most satisfactory and encouraging.

---

RED-TAPE has much to answer for, witness the case of a poor man at Leeds. Dukes had been employed as a navvy on the Yeadon and Guiseley Railway, and had been lodging at Guiseley. On April 16th he had his leg broken and received other injuries by the fall of some earth. He was taken to Leeds General Infirmary, and he remained in the institution until May 15th. Mr. Blair, the manager, then sent word to the relieving officer of the Leeds Union that all had been done for Dukes that could be done, and asked that, as he was without a home and friends, an order for his admittance to the workhouse should be granted. It was added that all the man required was rest. In reply, the following letter was sent: "Leeds Union, May 15th.—Re Wm. Dukes.—According to instructions which I have received, I regret having to refuse the above case.—Yours obediently, W. S. Carr." The infirmary authorities then asked what the reason was for the refusal, and a message was sent back to the effect that the infirmary authorities would have to retain Dukes, as they had taken him in; that as long as he was in the infirmary he was not destitute, and the union authorities could not therefore accept him. The infirmary authorities had then no other course open to them but to send the man to the Poor Law Offices in charge of one of the porters. He was left on the steps of the offices, and an intimation was given to the officers of the union that, now he was out of the infirmary, Dukes was in a state of destitution. It seemed that the man fainted whilst he was at the board's offices. The board of guardians has appointed a committee to discuss the whole question with the infirmary board. We hope this is consoling to poor William Dukes.

